# Pacbio Sequencing of PLC/PRF/5 Cell Line and Clearance of HBV Integration Through CRISPR/Cas-9 System

**DOI:** 10.3389/fmolb.2021.676957

**Published:** 2021-08-17

**Authors:** Chia-Chen Chen, Guiwen Guan, Xuewei Qi, Abudurexiti Abulaiti, Ting Zhang, Jia Liu, Fengmin Lu, Xiangmei Chen

**Affiliations:** ^1^ Department of Microbiology and Infectious Disease Center, School of Basic Medical Sciences, Peking University Health Science Center, Beijing, China; ^2^ Peking University People’s Hospital, Peking University Hepatology Institute, Beijing Key Laboratory of Hepatitis C and Immunotherapy for Liver Diseases, Beijing, China

**Keywords:** HBV integration, PLC/PRF/5, third-generation sequencing, pacbio, HBsAg clearance, CRISPR/Cas9

## Abstract

The integration of HBV DNA is one of the carcinogenic mechanisms of HBV. The clearance of HBV integration in hepatocyte is of great significance to cure chronic HBV infection and thereby prevent the occurrence of HBV-related hepatocellular carcinoma (HCC). However, the low throughput of traditional methods, such as Alu-PCR, results in low detecting sensitivity of HBV integration. Although the second-generation sequencing can obtain a large amount of sequencing data, but the sequencing fragments are extremely short, so it cannot fully explore the characteristics of HBV integration. In this study, we used the third-generation sequencing technology owning advantages both in sequencing length and in sequencing depth to analyze the HBV integration characteristics in PLC/PRF/5 cells comprehensively. A total of 4,142,311 cleaning reads was obtained, with an average length of 18,775.6 bp, of which 84 reads were fusion fragments of the HBV DNA and human genome. These 84 fragments located in seven chromosomes, including chr3, chr4, chr8, chr12, chr13, chr16, and chr17. We observed lots of DNA rearrangement both in the human genome and in HBV DNA fragments surrounding the HBV integration site, indicating the genome instability causing by HBV integration. By analyzing HBV integrated fragments of PLC/PRF/5 cells that can potentially express HBsAg, we selected three combinations of sgRNAs targeting the integrated fragments to knock them out with CRISPR/Cas9 system. We found that the sgRNA combinations could significantly decrease the level of HBsAg in the supernatant of PLC/PRF/5 cells, while accelerated cell proliferation. This study proved the effectiveness of third-generation sequencing to detect HBV integration, and provide a potential strategy to reach HBsAg clearance for chronic HBV infection patients, but the knock-out of HBV integration from human genome by CRISPR/Cas9 system may have a potential of carcinogenic risk.

## Introduction

Hepatocellular carcinoma (HCC) is one of the most aggressive human cancers which currently ranks the fourth leading cause of cancer-related deaths worldwide (Petrick et al., 2020). The major risk factors for HCC include infection with hepatitis B virus (HBV) and hepatitis C virus (HCV), exposure to aflatoxins, alcohol abuse, and non-alcoholic steatohepatitis (Baecker et al., 2018). The integration of HBV in the human genome is considered to be one of the key mechanisms of HBV carcinogenesis (D'Souza et al., 2020). However, the characteristics of integration, its functions, and the clinical implications are still not fully understood.

The detection of HBV integration is the basis of all HBV integration research. In the early 1980s, HBV DNA integration was firstly found in the human chromosome of PLC/PRF/5 cell line (Edman et al., 1980). Subsequently, a series of studies confirmed HBV DNA is randomly inserted into the human genome in HBV-infected liver tissues, including acute hepatitis B, chronic hepatitis B, cirrhosis and especially HCC (Jiang et al., 2012; Li et al., 2014). Previously, Southern blot, FISH (fluorescence *in situ* hybridization) and Alu-PCR have been used to detect HBV integration, but due to the low throughput, these methods are less sensitive. With the development of next-generation sequencing (NGS), many studies have achieved genome-wide surveys of HBV integration in HCC, and a great deal of data on the HBV pathogen in HCC has been obtained. However, NGS-based integration discovery is limited by the reads length, especially for long complex structural variations (Zhao et al., 2016a). The third-generation sequencing (TGS) technology developed in recent years not only has extremely high sequencing throughput, but also generate reads up to 60 kbp long (Rhoads and Au, 2015), showing a good potential for detecting HBV integration.

Studies have shown that the integrated HBV genome can express viral proteins, such as HBsAg and HBx (Natoli et al., 1992; Tu et al., 2001). Among them, HBsAg is not only a serological marker of HBV infection, but also associated with the occurrence of HCC. Because existing antiviral therapy does not target HBV integration, the integration of HBV sequence expression of HBsAg is also one of the reasons why CHB patients are difficult to achieve clinical cure (Moucari et al., 2009). Although studies have shown that siRNA can effectively knock down the HBsAg level in PLC/PRC/5 cell lines, this technology cannot eliminate the integrated HBV genome (Li et al., 2004). CRISPR/Cas (clustered regularly interspaced short palindromic repeat/CRISPR associated Cas) is considered a breakthrough technology of genome editing, mainly because of its high effectiveness and technical simplicity (Lin et al., 2014). The earlier results in our laboratory have confirmed that CRISPR/Cas9 technology can eliminate HBV cccDNA effectively, and we have designed a series of sgRNA targeting HBV genome (Wang et al., 2015; Wang et al., 2017), but whether it can effectively knock out HBV integration is yet to be revealed.

To gain a comprehensive panorama of HBV DNA integration of PLC/PRF/5 cell line in a more unbiased way, this study directly sequenced the DNA of PLC/PRF/5 cells using the whole genome TGS technology. In addition, the CRISPR/Cas9 technology was used to knock out the S genes in the HBV DNA integrated fragments, so as to inhibit HBsAg expression in PLC/PRF/5 cells. This study provides a more accurate and comprehensive information of HBV integration in PLC/PRF/5 cells and a new idea for the HBsAg clearance from HBV integration genome.

## Results

### Detection of Hepatitis B Virus Integration in PLC/PRF/5 Cell Line Using Third-Generation Sequencing Technology

**FIGURE 1:**
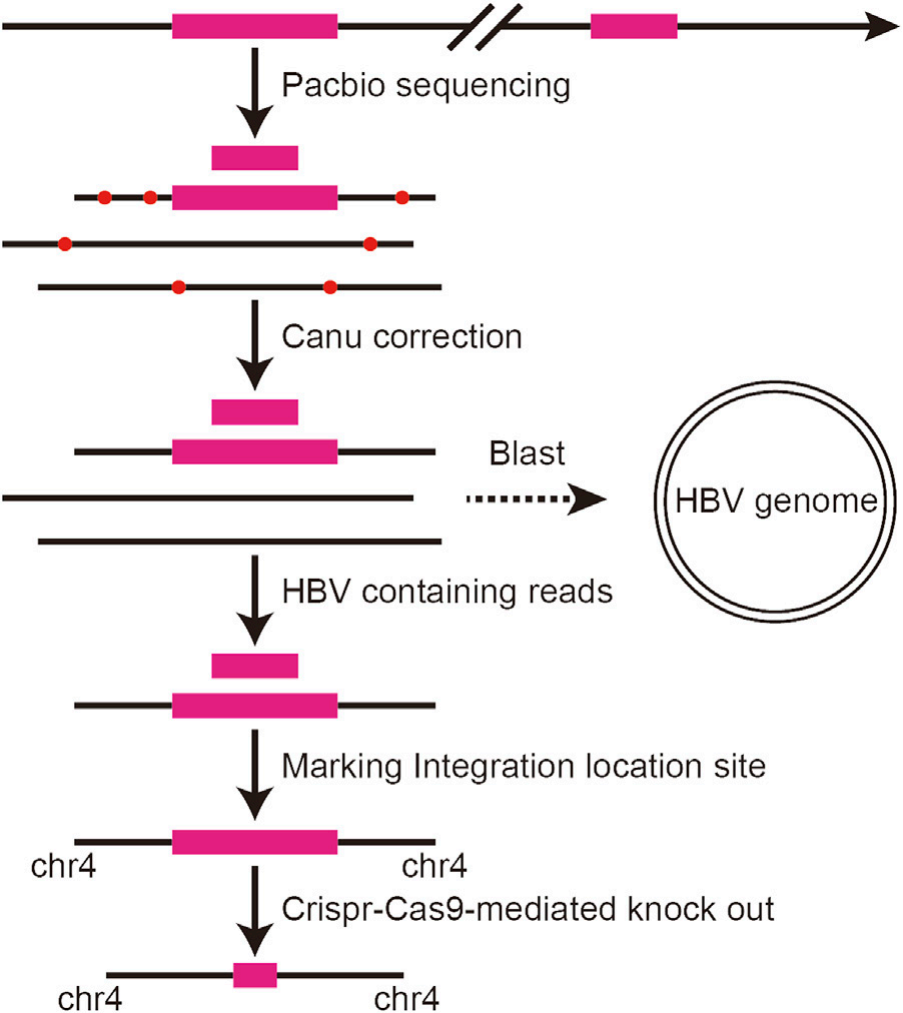
Analysis flow chart of HBV integration in PLC/PRF/5 cells.

We applied the third-generation sequencing technology to scan the HBV integration site at the whole genome level in PLC/PRF/5 cells, and the analysis flow chart is shown in Figure 1. In brief, the DNA extracted from PLC/PRF/5 is sequenced by the Pacbio platform, and then the correction module of the Canu software was used for row reads correction. Next, the reads were mapped to the HBV genome and the HBV containing reads were extracted by using of BLAST. Finally, the TSD software was used to analyze the HBV integration sites.

**FIGURE 2:**
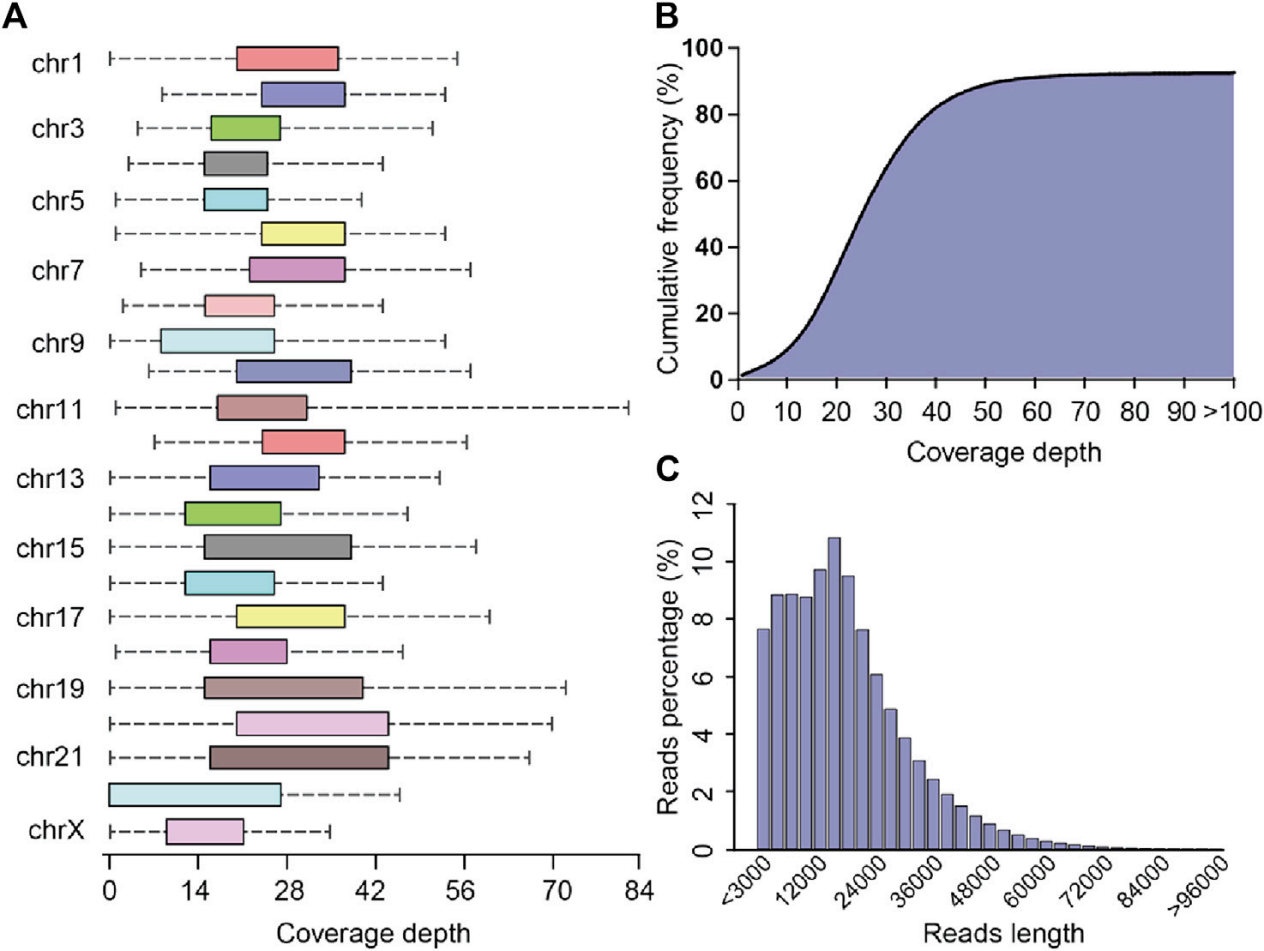
Sequencing coverage and reads length distribution plots **(A)** Box plot of sequencing coverage of each chromosome. **(B)** Cumulative percentage plots of coverage depth for the whole genome, the *x*-axis represents the coverage depth, and the *y*-axis represents the percentage of bases that reach this coverage depth level. **(C)** Distribution diagram of reads length.

The PLC/PRF/5 pacbio raw sequencing data contained 5,770,093 reads, totaling 99.3 billion bases, and the average sequencing depth was about 33.1 times. By the correction of Canu, we finally obtained 4,142,311 reads, totaling 77.7 billion bases, and the average sequencing depth was about 25.9 times. After mapping with the human reference genome, the coverage of cleaning reads in chr2, chr3, chr4, chr7, chr8, chr10, and chr12 reached 100%, while the coverage of chr22 was the lowest, about 71.7% (Figure 2A). Overall, the coverage of the whole genome by sequencing data reached 91.7%. (Figure 2B). The maximum length of the reads is 120445 bp, and the average reads length is 18,775.6 bp (Figure 2C). The above result shows that the quality and coverage of this sequencing reached the downstream analysis requirements.

### Hepatitis B Virus Integration Profiles in PLC/PRF/5 Cells

**FIGURE 3:**
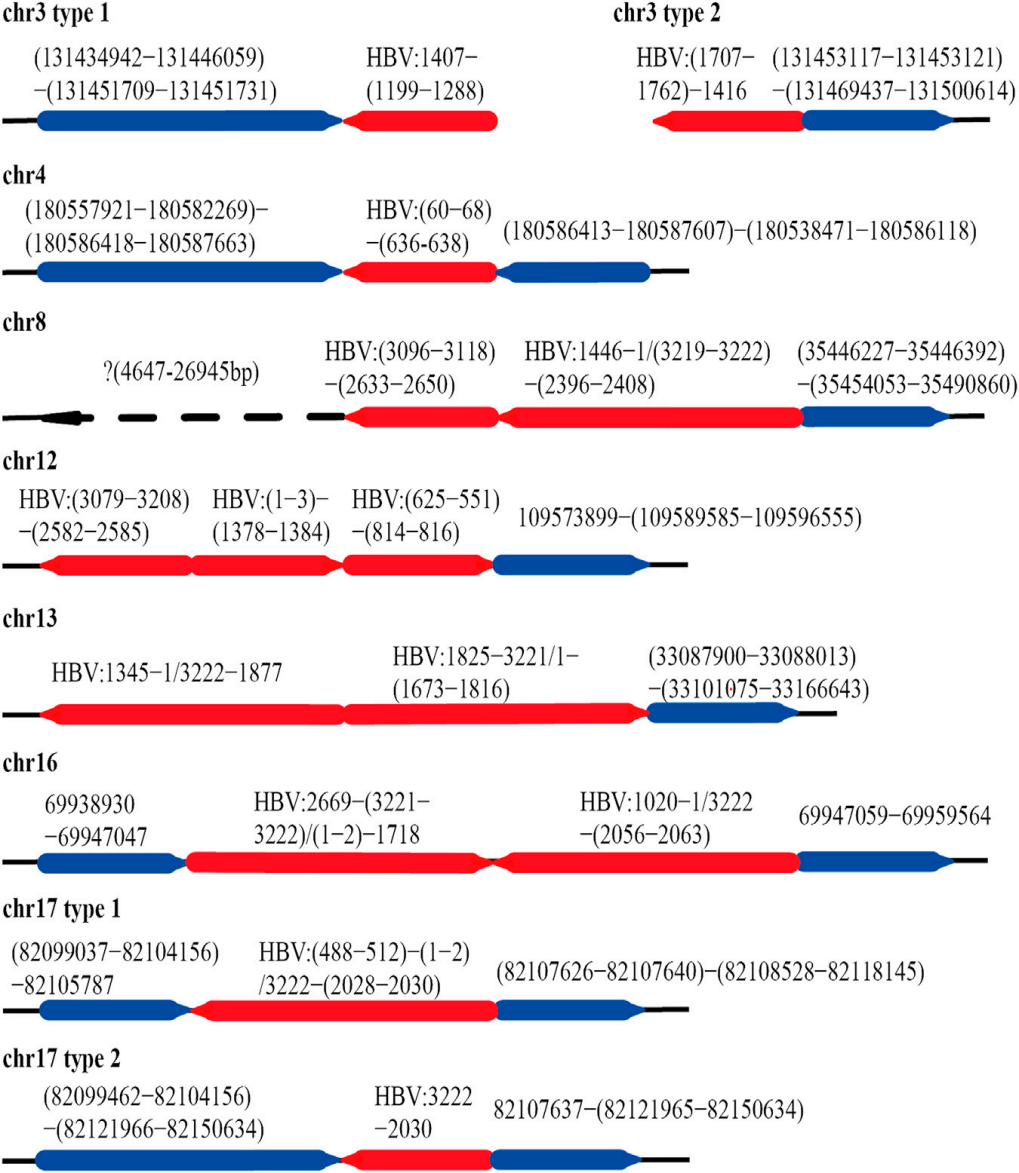
HBV integration pattern plots of PLC/PRF/5 cell line. The red line represents the HBV genome, the blue line represents the human genome, the direction of the arrow represents the direction of the sequence, and the dashed line represents the sequence not mapping to the human reference genome.

After mapping with HBV and the human genome, we obtained 84 HBV-human genome fusion reads in PLC/PRF/5 cell line, with reads length range 5,067–119,485 bp (SupplementaryTable S1). These reads are located on 7 chromosomes including chr3, chr4, chr8, chr12, chr13, chr16 and chr17 (Figure 3). The reads located at chr4, chr8, chr16 and chr17 contain at least one read that both the 3 end and 5 end are human genome thereby we called it complete sequence. For the reads located on chr3, although we did not find the complete sequence, we found two types of reads, one is HBV fragment at the 3 end and the other is HBV fragment at the 5 end. Therefore, for the above five chromosomes, we confirmed both the upstream and downstream integration sites of HBV DNA. As for chr12 and chr13, we can only confirm the downstream site of HBV integration.

The integration of HBV often causes genome instability. Previous studies could not fully explore this problem due to the limitation of sequencing length. Based on the advantages of the third-generation sequencing length, we comprehensively analyzed the instability of HBV integration fragment and the nearby human genome. In chr3 and chr16, we observed about 1400 and 12 bp human genome fragment deletions near the HBV integration site, respectively (Figure 3; Supplementary Figure S1). In chr4, a large-scale genome amplification was observed, and the amplified fragments of each reads are not the same. It is worth noting that in chr4, the insertion direction of the copy number amplified fragment is opposite to the original direction of the genome (Figure 3; Supplementary Figure S1). In chr17, we found two types of reads, one of which contained about 1800 bp human genome deletion near the insertion site, and another type contained about 14600 bp human genome amplification (Figure 3; Supplementary Figure S2). In chr8, the sequence upstream of the HBV insertion site cannot be mapped to the human genome. The sequence similarity comparison shows that the homology of this fragment with chr8 is 72%, and the homology with chr1 is 79% (Figure 3). In addition, viral genome rearrangements including deletions, inversions and duplications were also observed. For example, the end of the integration fragment in chr12 is located at positions 814 nt–816 nt of the HBV genome, with a difference of 3 bp, and the end of the integration fragment in chr13 is located at 1673 nt–1799 nt of the HBV genomes, with a difference of more than 100 bp. In chr17, there is a big difference in the length of the HBV integration fragment, the shortest one is 1192 bp, and the longest one is 1706 bp (Figure 3; Supplementary Figure S2). The above results indicate that the human genome and HBV genome nearby the integration site contain many structural variations, suggesting that HBV integration increases the genome instabilities in both the HBV integrated fragment and the nearby human genome.

### Distribution of Hepatitis B Virus Integration Fragment Breakpoints and Its Homology With Human Chromosomes

**FIGURE 4:**
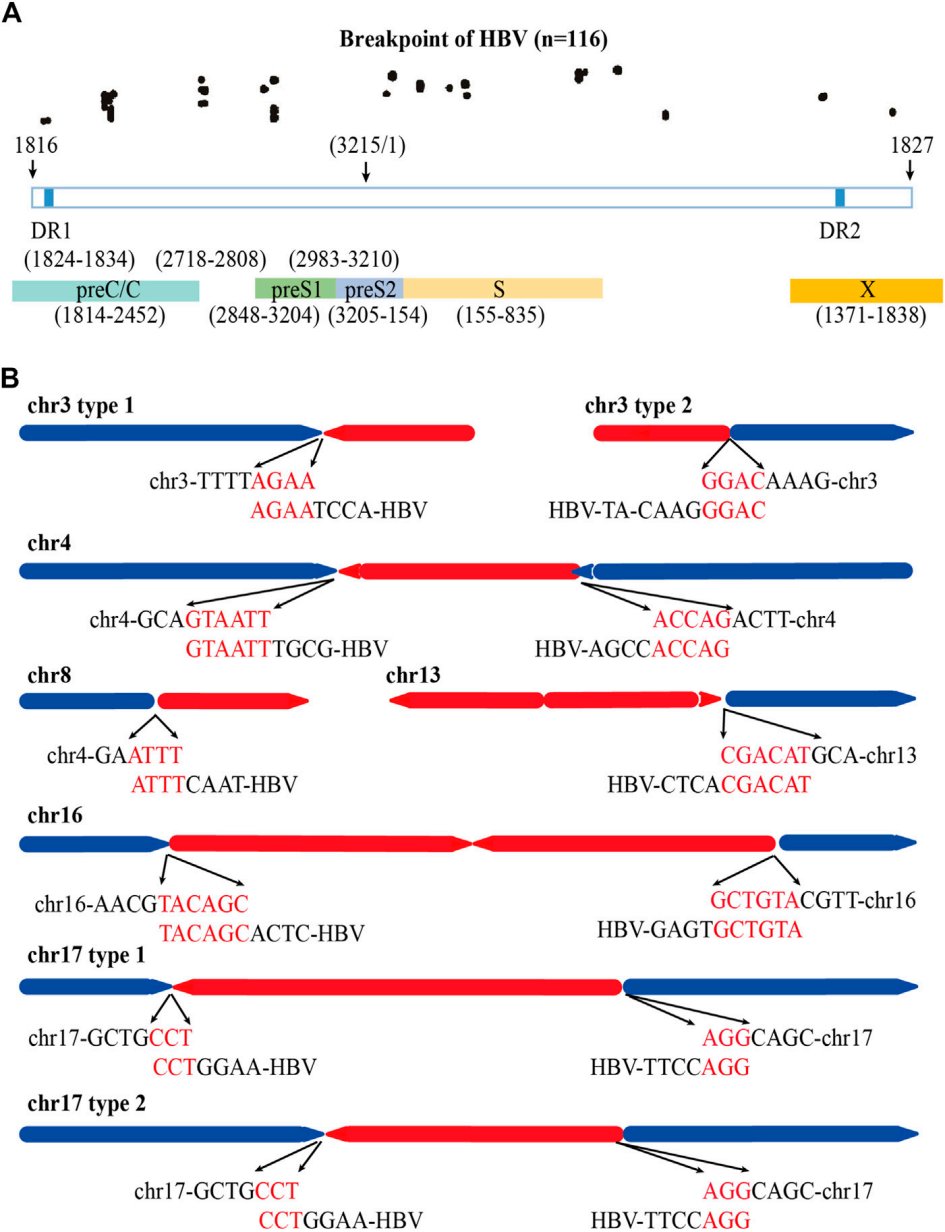
Distribution of HBV integration fragment breakpoints and its homology with human chromosomes. **(A)** Distribution of HBV integration fragment breakpoints in HBV genome. **(B)** Homology analysis of virus-host junction sequences in HBV integration events. The red line represents the HBV genome, the blue line represents the human genome, the direction of the arrow represents the direction of the sequence, and the red words represents microhomology connections of HBV DNA and human genome.

Previous study shows that HBV breaks mainly at the DR1 and DR2 regions and integrates into human genome (Li et al., 2014). We also analyzed the distribution of the HBV integration breakpoints of the 84 HBV containing reads in PLC/PRF/5 cells. A total of 116 HBV breakpoints were identified and the distribution of these breakpoints is shown in Figure 4A. We found that rather than concentrated in the DR1 and DR2 regions, the HBV breakpoints of PLC/PRF/5 distributed randomly throughout the HBV genome.

Next, we explored the homology of the HBV genome and sequencing of the host cell genome close to the viral-host junction revealed 52 (44.8%) microhomology connections among 116 HBV breakpoints between the HBV fragment and the human genome in chr3, chr4, chr8, chr13, and chr17. Among them, the micro homologous sequences in chr3 are AGAA and GGAC, in chr4 are GTAATT and ACCAG, in chr8 are ATTT, in chr13 are CGACAT, in chr16 are TACAGC and GCTGTA, and in chr17 are CCT and AGG (Figure 4B).

### Host Genes Around the Hepatitis B Virus Integration Sites

Previous studies by us and other teams have confirmed that HBV integration can affect the expression of genes near the integration site through a variety of mechanisms, which is one of the carcinogenic mechanisms of HBV integration (D'Souza et al., 2020). We analyzed the genes within 100 kb upstream and downstream of the HBV integration sites in PLC/PRF/5 cells. There are 27 genes in the upstream of HBV integration sites and 13 genes in the downstream of HBV integration sites, with a total of 43 potential target genes. Among the 43 genes, 23 genes were protein coding genes, 9 genes were non-coding RNA, 3 genes were miRNA and 8 genes were pseudogenes (SupplementaryTable S2). This result suggested that there were a large number of host functional genes around HBV integration sites that might be affected by HBV integration.

### Expression Potential of Viral Proteins by the Integrated Hepatitis B Virus Fragment

**FIGURE 5:**
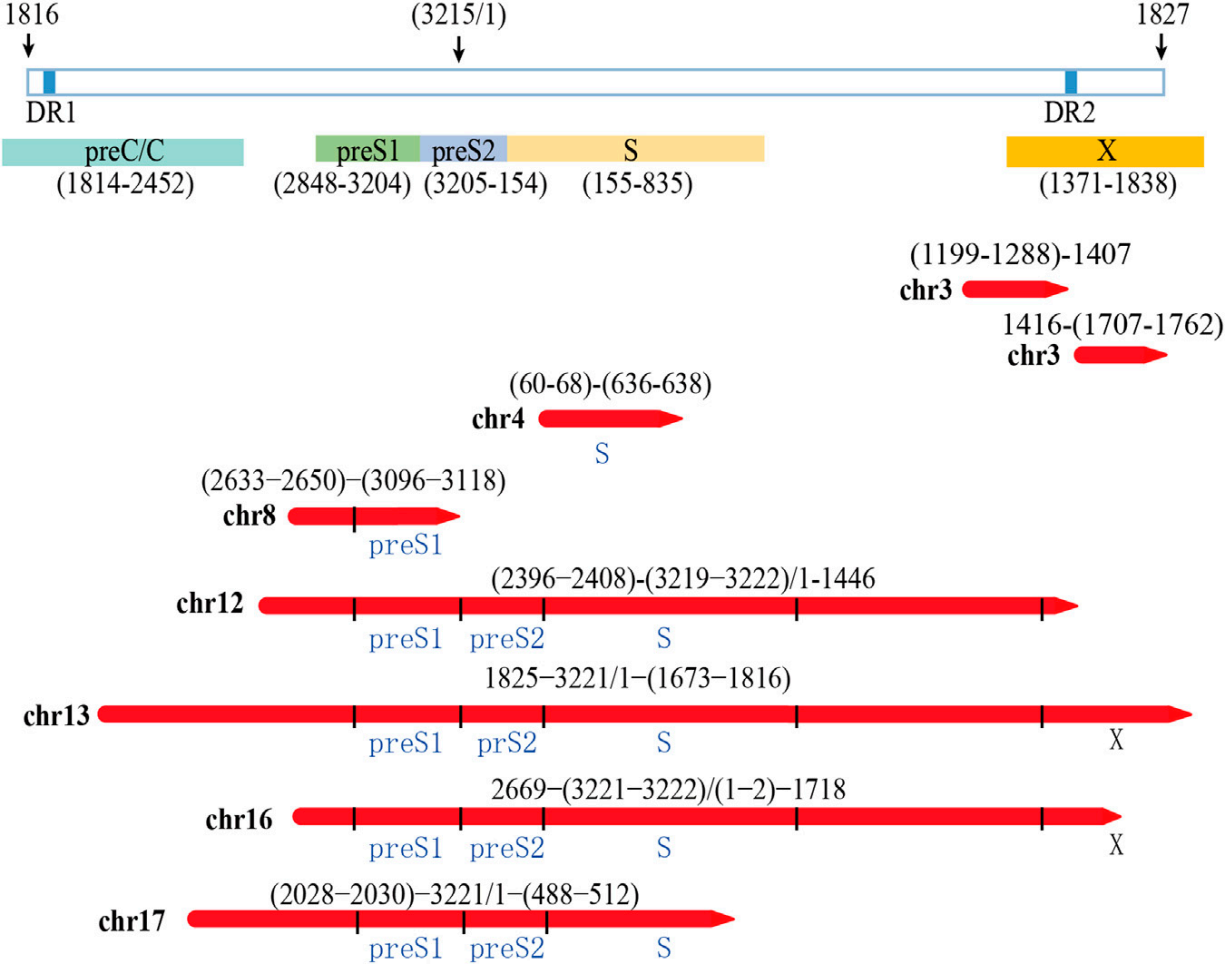
Analysis of the possibility of integrating HBV fragment sequences expressing HBV viral proteins

The integrated HBV fragments have the potential to express HBV proteins or HBV-human fusion proteins (Edman et al., 1980; D'Souza et al., 2020;). According to the sequencing results, we analyzed the possible viral proteins expressed by the integrated fragments in PLC/PRF/5 cells. The analysis results showed that the integrated fragments located in chr12, chr13, chr16, and chr17 have the potential to express HBsAg. Moreover, the integrated fragments located in chr13 and chr16 also have the potential to express truncated HBx (Figure 5). In consistent with these analyses, we can detect the expression of HBsAg in the supernatant of PLC/PRF/5 cells, which reached 1.796 IU/mL.

### Knock-Out of Integrated Hepatitis B Virus Genes by CRISPR/Cas9 System in PLC/PRF-5 Cells

Our previous studies have designed 15 gRNAs against HBV genome and demonstrated these gRNAs could specifically destroy HBV expressing template, thereby inhibited HBV replication (Wang et al., 2015). To eliminate HBsAg in PLC/PRF/5 cell lines, we selected sgRNA2, sgRNA3 and sgRNA7 from our previous studies and the targeting sites of these sgRNA in integrated HBV fragments are shown in Supplementary Figure S3. For better knocking out the integrated preS/S gene fragments in the PLC/PRF/5 cell genome, the HBV specific gRNA/Cas9 plasmids containing sgRNA2, sgRNA3 and sgRNA7 were combined in pairs for transient transfection. To determine whether the paired gRNA can successfully knock out the target HBV integration fragments, two pairs of PCR primers were designed respectively in the upstream and downstream of sgRNA, and the target sites of the primer are shown in the Supplementary Figure S3.

**FIGURE 6:**
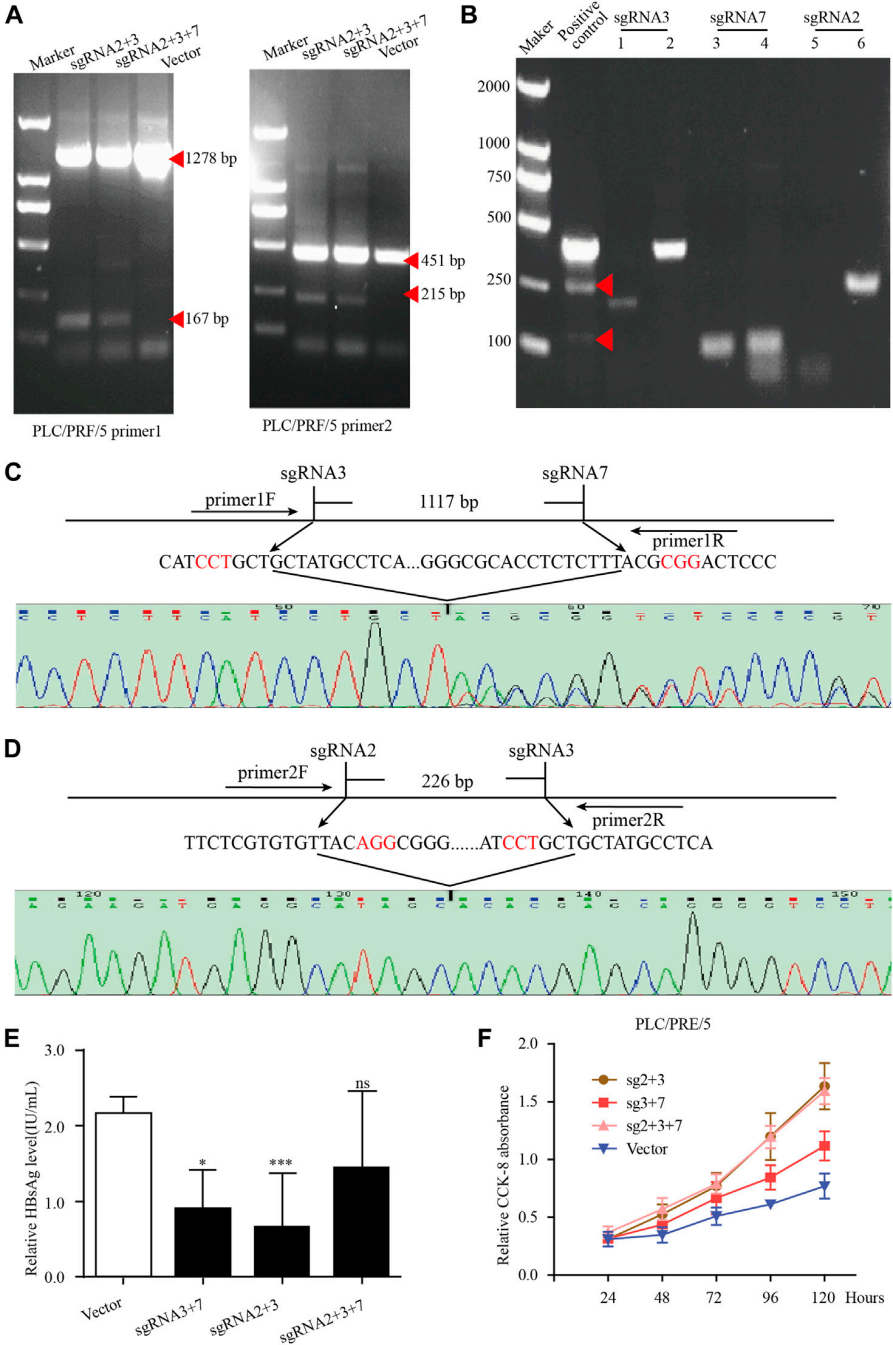
The knockout of integrated HBV S gene by CRISPR/Cas9 system in PLC/PRF/5 cells. **(A)** The plasmid pBB4.5-HBV1.2 (0.5 μg) was co-transfected with gRNA2+3, gRNA3+7 and gRNA2+3+7 expression vectors (each 0.75 μg) to PLC/PRF/5 cells. Cellular DNA was extracted at 72 h post transfection, and PCR amplifications were performed using the primers beyond the cleavage sites of each dual gRNAs. **(B)** The off-target effect of sgRNA was detected by T7 endonuclease I assay. Each sgRNA detected the two most likely off-target sites. **(C–D)** Sequencing analysis of the smaller fragment formed by sgRNA3+7 and sgRNA2+3. **(E)**. HBsAg level in culture supernatant was measured by using an enzyme-linked immune sorbent assay. Data are shown as mean ± SE of 3 independent samples. Statistical method: *t* test (two-side), * means *p* < 0.05, ** means *p* < 0.01,*** means *p* < 0.001. **(F)** The cell proliferation of PLC/PRF/5 cells after transfecting different sgRNA plasmids. Cells transfected with empty PX458 plasmid were used as control group. Data are shown as mean ± SE of 3 independent samples. Statistical method: *t* test (two-side).

In PLC/PRF/5 cells, after transfection of HBV specific gRNA/Cas9 plasmids expressing sgRNA2+3, sgRNA3+7 and sgRNA2+3 + 7, the expected small bands were observed by using PCR assay (Figure 6A). No obvious off-target effects were observed in the predicted off-target sites corresponding to the three sgRNAs by using the T7 endonuclease I assay (Figure 6B). The sequencing of these small bands showed that the integrated HBV fragments were indeed cut at the expected sites and the integrated HBV fragments were knocked out (Figures 6C,D). Most importantly, the HBsAg level in the supernatant was significantly decreased in these PLC/PRF/5 cells with integrated S genes knocked-out (Figure 6E). Unexpectedly, we noticed that no matter which sgRNA combination, after sgRNAs transfection, the proliferation rate of PLC/PRF/5 cells was significantly faster than that of the control group (Figure 6F). We also performed the same knock-out assays with same sgRNA combination together with 1.2 mer HBV plasmid in Huh7 cells. We found that the sgRNA combination could significantly knocked out HBV S gene and downregulated HBsAg level in Huh7 cells, demonstrating that the combined HBV specific gRNA/Cas9 plasmids could efficiently decline HBV expressing templates (Supplementary Figure S4). Taken together, these results suggested that CRISPR/Cas9 system could effectively knock out the S gene from the integrated genome, but it might accelerate cell proliferation of hepatocytes.

## Discussion

HBV integration is regarded as a pivotal process of HBV infection to induce liver cancer. In this study, we detected the HBV integration profiles at the whole genome level in PLC/PRF/5 cells by using the whole genome TGS technology. This method is a novel technique that overcomes the low-throughput shortcomings of sanger sequencing and also overcomes the short reads shortcomings of the NGS. Our sequencing data will help researchers to have a more comprehensive understanding of the genome characteristics of PLC/PRF/5, the most classic cell model for studying HBV integration. In addition, using CRISPR/Cas9 system, we knocked out the integrated HBV S fragment and decreased HBsAg level in PLC/PRF/5 cells, suggested the potential use of CRISPR/Cas9 genome editing technique to cut integrated HBV DNA, as well as for function cure of chronic hepatitis B patients.

As early as the 1980, researchers have found that PLC/PRF/5 cell line genome contains HBV genome integration (Edman et al., 1980). Since then, the HBV integration of PLC/PRF/5 cell line had been successively detected by Northern blot, FISH, Alu-PCR and NGS technology (Jiang et al., 2012; Li et al., 2013; Watanabe et al., 2015; Ishii et al., 2020). Although researchers used the HBV-specific probe capture technology to improve the sensitivity of HBV DNA detection and found that PLC/PRF/5 has HBV integration on chromosomes 3, 4, 5, 8, 10, 11, 12, 13, 16, 17, 19 (Li et al., 2013; Watanabe et al., 2015; Ishii et al., 2020), there were selective bias in this technology so that it is impossible to have a comprehensive analysis of the characteristics of PLC/PRF/5 HBV integration. Meanwhile, it is hard to analyze the complex variation of the genome sequence surrounding the HBV integration sites through NGS because the length of the sequencing fragment was only 100 bp. In this study, we directly sequenced the DNA of PLC/PRF/5 cell which made the maximum length of the sequencing segment reach 150 kbp. The longest HBV integrated fragment detected in this study was as long as 40 kbp. Therefore, the results of this study can not only detect HBV integration in PLC/PRF/5 cell lines without bias but also made an in-depth analysis of the structural variation of the upper and lower host genome. We successfully detected HBV integration on chromosomes 3, 4, 8, 12, 13, 16, and 17 in PLC/PRF/5 cell line through the whole genome TGS technology. From the perspective of detection sensitivity, our method is much higher than Northern blot, FISH, Alu-PCR, and even has the same sensitivity as the targeted captured NGS technology, proving that the whole genome TGS technology is a powerful method for detecting the HBV integration.

Studies have shown that HBV integration affects the genetic stability near the integration site, which is one of the reasons for HBV integration to cause cancer (Sung et al., 2012). Since the whole genome TGS technology can obtain longer integration fragments, it is more suitable for the analysis of rearrangement of integrated HBV sequence and the local host genome sequence surrounding integration sites. In this study, we found that there are many viral DNA rearrangements in the integrant including deletions, inversions and duplications of HBV sequence. In addition, sequences of host genome near the viral-host junction also has also undergone structural changes. Additionally, despite the fact that previous studies have showed the breakpoint of HBV integration concentrated between DR1 and DR2 (Li et al., 2014) due to the features of dslDNA which is the major source of the HBV integration (Tu et al., 2017), in this study, we observed that the breakpoints of HBV integration in PLC/PRF/5 cell lines are randomly distributed. All these results indicate that HBV integration will lead to the instability of the genome near the integration site which was contributed to the pathogenesis of HCC, while the long-term host DNA repair during tumor cell proliferation exacerbate the genetic instability in host cells. Besides genetic instability, we also observed that about 45% of the viral-host junctions sites in PLC/PRF/5 cell connect to the human genome were microhomology connections contained a short homologous region between HBV integration fragments and the human genome sequences. It is consisted with the previous studies that proposed homologous end joining is the major source for HBV to integrate into the human genome (Zhao et al., 2016b) (Zhao et al., 2016b) (Zhao et al., 2016b) (Zhao et al., 2016b).

In recent years, quantitated serum HBsAg has been becoming an important viral marker for evaluating the response to antiviral therapy, and the seroclearance of HBsAg is regarded as the most reliable indicator for functional cure by several major guidelines (Sarin et al., 2016). A large number of studies have confirmed that the HBV sequence integrated into the genome has the potential to express HBV protein (Sung et al., 2012). Therefore, serum HBsAg can be produced from both HBV cccDNA and the integrated HBV S gene in patients with HBV integration. Especially in the HBeAg-negative group, the main source of serum HBsAg was mainly derived from integrated HBV fragments (Wooddell et al., 2017). Because the current antiviral drugs could not eliminate HBsAg production originated from the integrated HBV S gene, it is very difficult to obtain serum HBsAg loss even if prolonging nucleos(t)ide analogue (NUC) therapy. It has been demonstrated that CHB patients who achieve HBsAg seroclearance have reduced risks of HCC development (Liu et al., 2016). Therefore, effective strategies to eliminate integrated HBV DNA fragment-derived HBsAg should be developed. In PLC/PRF/5 cells, we found that the integration of HBV located in chr13, chr16, and chr17 in lines has the potential to express HBsAg. Based on the sequences of these integrated HBV DNA fragments, we selected three CRISPR/Cas9-sgRNA combinations to target HBV S gene in PLC/PRF/5 cells. The results showed that all our CRISPR/Cas9-sgRNA combinations could effectively reduce the HBsAg level in the PLC/PRF/5 cell supernatant, proving that CRISPR/Cas9 system could effectively eliminate HBV integration. The previous results in our laboratory have confirmed that the transfection of these CRISPR/Cas9-sgRNA plasmid did not affect the proliferation rate of Huh-7 cells when co-transfecting HBV plasmids (Wang et al., 2017). Surprisingly, in this study we found that the proliferation rate of PLC/PRF/5 cells increased significantly after transfection of the three CRISPR/Cas9-sgRNA combinations, suggested that the potential dangers of CRISPR/Cas9 need to be carefully considered.

We speculate that the cleavage of chromosomally integrated HBV DNA may trigger unpredictable consequences of chromosomal DNA recombination which induce cell proliferation. In addition, there are a large number of host functional genes located within 100 kb upstream and downstream of the HBV integration sites in PLC/PRF/5 cells. Whether these genes could be affected by the cut of integrated HBV DNA and thereby accelerate PLC/PRF/5 cell proliferation need be further explored.

In summary, our study provided a comprehensive panorama of HBV DNA integration profile of PLC/PRF/5 cell line, which is of great significance for us to fully understand the characteristics and mechanisms of HBV integration. In addition, under the guidance of the sequencing results, we successfully reduced the HBsAg level of PLC/PRF/5 through the CRISPR/Cas9 system, suggested that the cleavage of chromosomally integrated HBV DNA might be a new therapeutic approach for the clearance of HBsAg for CHB patients. However, the potential dangers of this approach must be further explored.

## Materials and Methods

### The Third-Generation Sequencing and Data Analysis

DNA was extracted from PLC/PRF/5 cell line using DNeasy Plant Mini Kit (Qiagen, Dusseldorf, Germany) according to the manufacturer's instructions and the quality of the extracted DNA was evaluated by agarose gel electrophoresis. Then DNA was commissioned to conduct subsequent DNA quality assessment, library construction, and third-generation sequencing by Annoroad Inc (Beijing, China). The sequencing platform was PacBio Sequel II. Through SMRTLink (v4.0) we converted the BAM format files into fastq format files. Next, the Canu (v2.0, default parameter) software (Koren et al., 2017) was used to clean and correct the fastq files. The coverage statistics of the sequencing were completed using the stats module of samtools (v1.9, default parameter) (Li et al., 2009). The cleaned and corrected reads were mapped with HBV A genotype by BLAST to obtain HBV containing reads. TSD software (Meng et al., 2019) was used to analysis the HBV integration site and hg19 was used as the reference genome. The genes around 100 kb upstream and downstream of the HBV integration site were searched on the NCBI website (https://www.ncbi.nlm.nih.gov/).

### Plasmids and Transfection

The 1.2xHBV plasmid was constructed using a 1.2-fold length HBV genome and pBB4.5 plasmid. The gRNA/Cas9 dual expression vector pSpCas9(BB)-2A-GFP (PX458) was obtained from Addgene (Cambridge, MA). The oligonucleotide sequences for the construction of HBV-specific gRNA/Cas9 expression vectors are listed in SupplementaryTable S3. The construction method was described in the previous research of our lab (Wang et al., 2015).

PLC/PRF/5 and Huh-7 cells were maintained in Dulbecco’s Modified Eagle Medium (DMEM) supplemented with 10% fetal bovine serum (Gibco, Maryland, United States). Before transfection, cells were seeded into a 12-well plate at 1.5 × 10^5^ cells/well. 24 h later, Huh-7 cells were co-transfected with HBV expression plasmid and HBV-specific gRNA/Cas9 dual expression plasmids with Lipofectamine 2000 (Invitrogen, New York, United States) for 72 h. PLC/PRF/5 cells were transfected with HBV specific gRNA/Cas9 dual expression plasmids with Lipofectamine 2000 for 72 h.

### Testing the Deletion of the Integrated Hepatitis B Virus S Gene Induced by CRISPR/Cas9

After the CRISPR/Cas9 plasmid transfecting, genomic DNA from PLC/PRF/5 or Huh7 cells were amplified by PCR using unique primers designed to span the expected knockout positions and the primers are listed in SupplementaryTable S4. The PCR products were gel purified and then sequenced by conventional Sanger sequencing. Off-target sites were predicted using website http://crispr.mit.edu/ and identified with T7E1 assay system as described in our previous studies (Liang et al., 2015). Primers for amplifying three sgRNAs off-target sites were shown in SupplementaryTable S5.

### Detection of HBsAg

Cell culture supernatants were collected for detection of HBsAg by a time-resolved fluoroimmunoassay according to manufacturer’s instructions (PerkinElmer, Waltham, MA). In brief, culture supernatant (100 μl) was added into a microtiter plate coated with anti-HBsAg and shook for 40 min at room temperature, then washed for four times. Europium-labeled anti-HBsAg was diluted 1:50 with HBsAg or HBeAg dilution buffer and added at 100 μl per well, shook for 40 min in room temperature, then washed six times. At last, after incubation with enhancement solution (100 μl) for 5 min, the plates were read using Anytest reader (SYM-BIO, Washington, United States), and the concentrations of HBsAg were calculated according to the standard curve. The relative HBsAg level was calculated as the ratio of HBsAg concentration in the cell culture supernatant of gRNA treated and vector control cells.

### Cell Counting Kit-8 Cell Proliferation Assay

Cell viability was evaluated using the Cell Counting Kit-8 (CCK8) assay (Dojindo, Japan) according to the manufacturer instruction. Cells were plated at a density of 1 × 10^3^ cells per well in 96-well plates for culturing 1, 2, 3, 4 and 5 days. At the indicated times, 10 μl CCK8 solution was added to each well and incubated at 37°C temperature. The absorbance was assessed at a 450 nm wavelength under a plate reader (Bio-Rad Laboratories, California, United States) after 2 h. All experiments were performed in triplicate.

### Statistical Analysis

The difference comparison of quantitative data is done by *t*-test using GraphPad Prism 5 software. *p*-value that less than 0.05 was considered statistically significant.

## Data Availability

The data presented in the study are deposited in the SRA (www.ncbi.nlm.nih.gov) repository, accession number (PRJNA717995).
