# Supplementary material for: Pacbio Sequencing of PLC/PRF/5 Cell Line and Clearance of HBV Integration Through CRISPR/Cas-9 System
Source: Front Mol Biosci. 2021 Aug 17;8:676957. doi: 10.3389/fmolb.2021.676957 (PMC8416172; doi:10.3389/fmolb.2021.676957)
Supplement: Supplementary file 1 [file DataSheet1.docx]

## Supplementary Figures


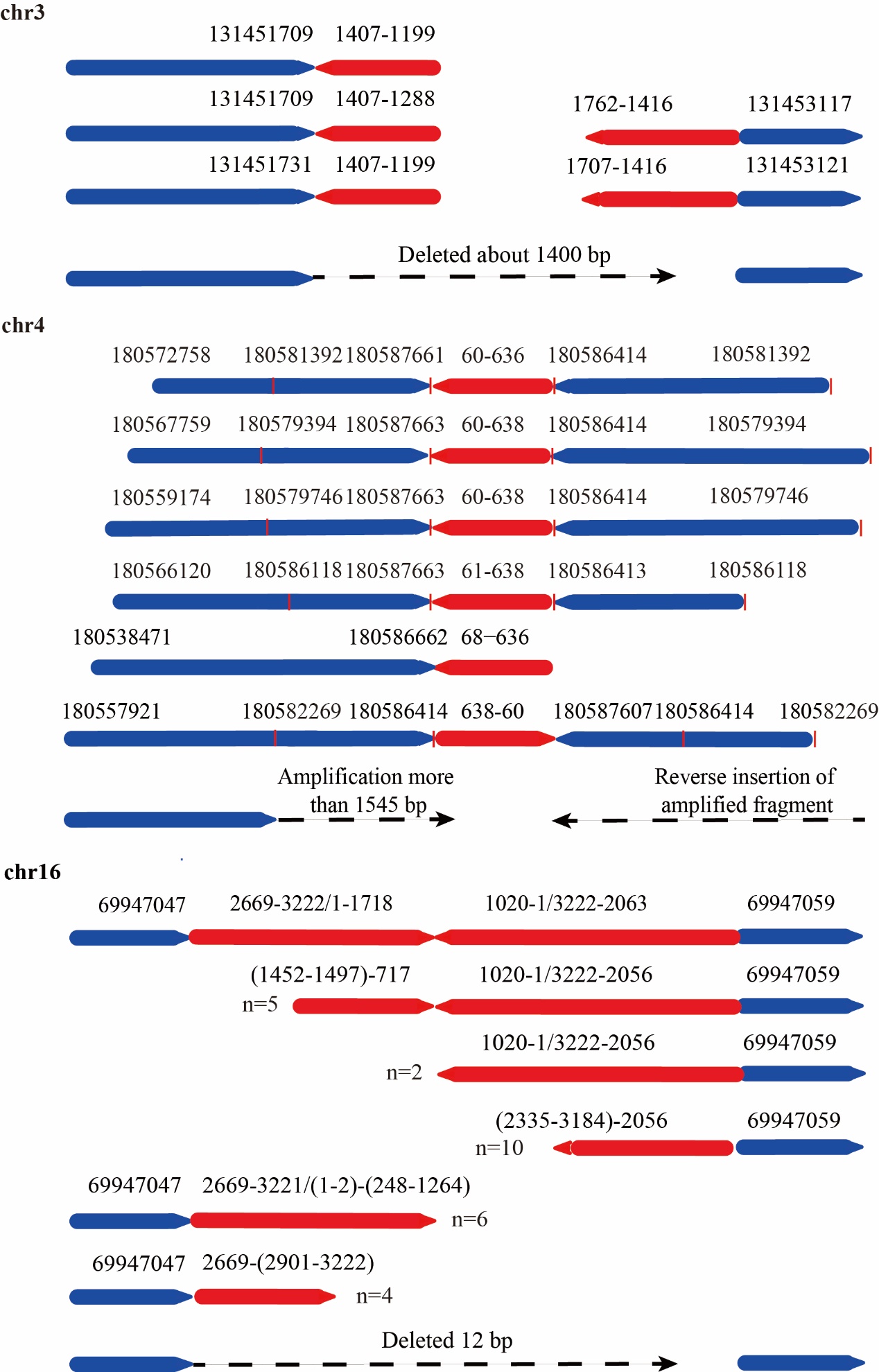


**Supplementary Figure 1.** **The copy number variation nearby HBV integration site in chr3, chr4 and chr16.**

The red line represents the HBV genome, the blue line represents the human genome, the direction of the arrow represents the direction of the sequence, the dashed line represents copy number variation of human genome, and the number of reads indicates the total number of reads of this type obtained by this sequencing.


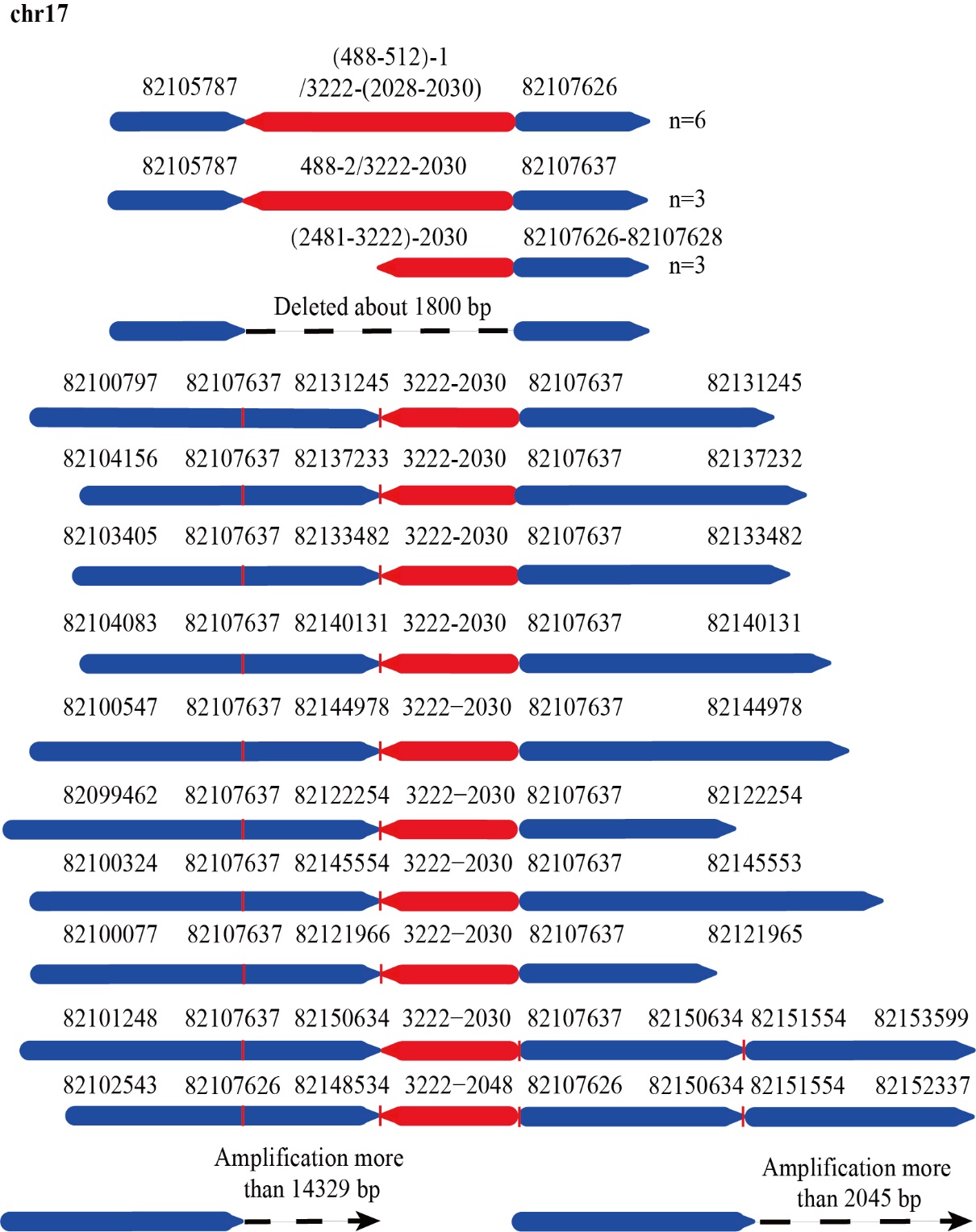


**Supplementary Figure 2: The copy number variation nearby HBV integration site in chr17.**

The red line represents the HBV genome, the blue line represents the human genome, the direction of the arrow represents the direction of the sequence, the dashed line represents copy number variation of human genome, and the number of reads indicates the total number of reads of this type obtained by this sequencing.


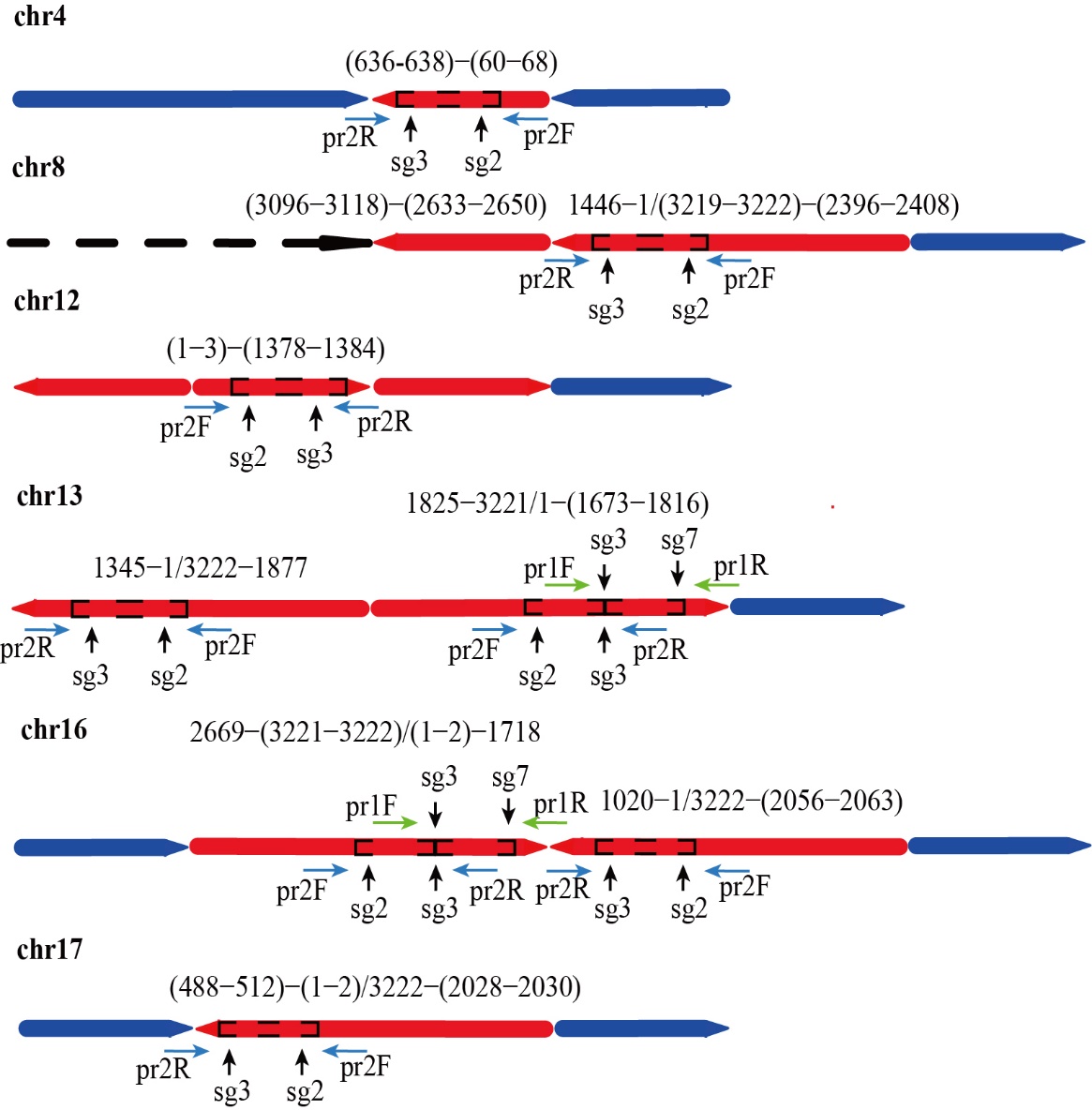


**Supplementary Figure 3: Target of sgRNAs and primers.**

The red line represents the HBV genome, the blue line represents the human genome, the direction of the arrow represents the direction of the sequence, the dashed line represents copy number variation of human genome, and the targets of sgRNA and primers are shown by arrows.


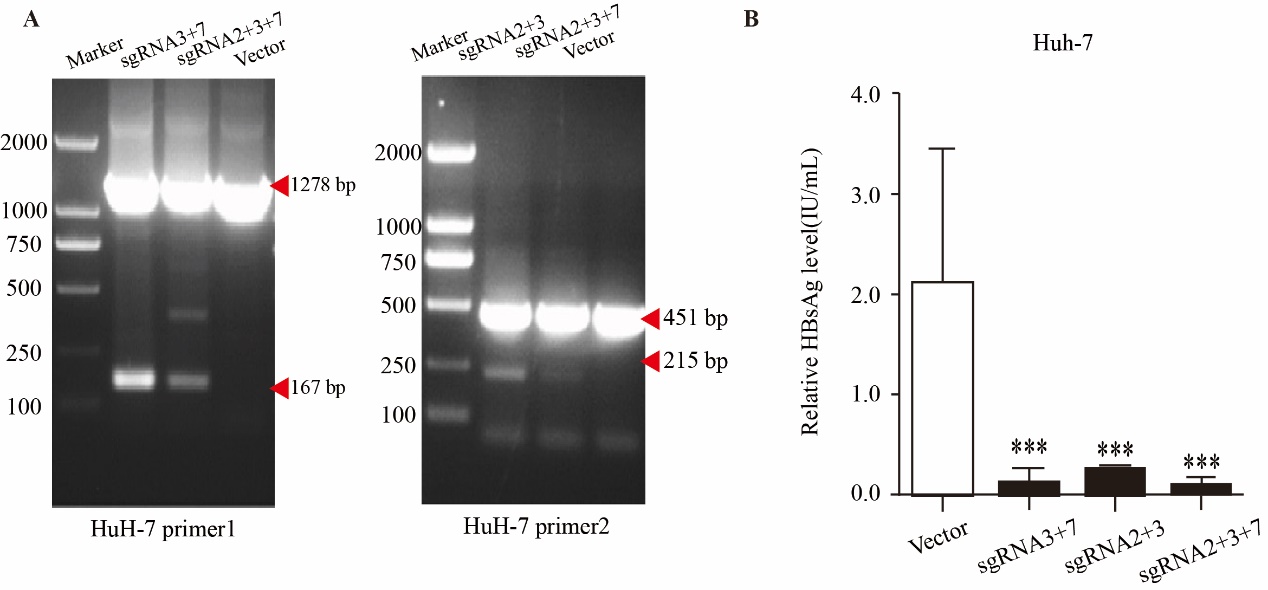


**Supplementary Figure 4: Confirmation of knockout efficiency of paired sgRNA by PCR, cell supernatant HBsAg quantification.**

(A) The plasmid pBB4.5-HBV1.2 (0.5 μg) was co-transfected with gRNA2 and 3, gRNA3 and 7, gRNA2, 3 and 7 expression vectors (each 0.75 μg) to HuH-7 cells. Cellular DNA was extracted at 72 h post transfection, and PCR amplifications were performed using the primers beyond the cleavage sites of each dual gRNAs.

(B) HBsAg level in culture supernatant was measured by using an enzyme-linked immune sorbent assay in Huh-7 cell line. Data are shown as mean ± SE of 3 independent experiments. The arrows mean that the digested fragment of the HBV expressing templates.
